# Author Correction: Caspase-8 mediates inflammation and disease in rodent malaria

**DOI:** 10.1038/s41467-020-19620-0

**Published:** 2020-11-03

**Authors:** Larissa M. N. Pereira, Patrícia A. Assis, Natalia M. de Araújo, Danielle F. Durso, Caroline Junqueira, Marco Antônio Ataíde, Dhelio B. Pereira, Egil Lien, Katherine A. Fitzgerald, Dario S. Zamboni, Douglas T. Golenbock, Ricardo T. Gazzinelli

**Affiliations:** 1 Instituto Rene Rachou, FIOCRUZ-MG, Belo Horizonte, MG 30190-002 Brazil; 2 Departamento de Bioquímica e Imunologia, ICB, Universidade Federal de Minas Gerais, Belo Horizonte, MG 31270-901 Brazil; 3 Department of Medicine, University of Massachusetts Medical School, Worcester, MA 01605 USA; 4 Centro de Pesquisas em Medicina Tropical, FIOCRUZ-RO, Porto Velho, RO 76812-329 Brazil; 5 Departamento de Biologia Celular Molecular e Bioagentes Patogenicos, Faculdade de Medicina de Ribeirão Preto, Universidade de São Paulo, Ribeirão Preto, SP 14049-900 Brazil; 6 Plataforma de Medicina Translacional, Fundação Oswaldo Cruz/Faculdade de Medicina de Ribeirão Preto, Ribeirão Preto, SP 14049-900 Brazil

**Keywords:** Infectious diseases, Inflammation, Parasite host response, Malaria

Correction to: *Nature Communications* 10.1038/s41467-020-18295-x, published online 14 September 2020.

The original version of this Article omitted the following from the Acknowledgements:

Fundação de Amparo a Pesquisa de São Paulo (Fapesp, 2016/23618-8).

This has now been corrected in both the PDF and HTML versions of the Article.

