# Supplementary material for: Caspase-8 mediates inflammation and disease in rodent malaria
Source: Nat Commun. 2020 Sep 14;11:4596. doi: 10.1038/s41467-020-18295-x (PMC7490701; doi:10.1038/s41467-020-18295-x)
Supplement: Supplementary file 1 — Supplementary Information [file 41467_2020_18295_MOESM1_ESM.pdf]

Supplementary Information

Caspase-8 mediates inflammation and disease in rodent malaria

Pereira et al.

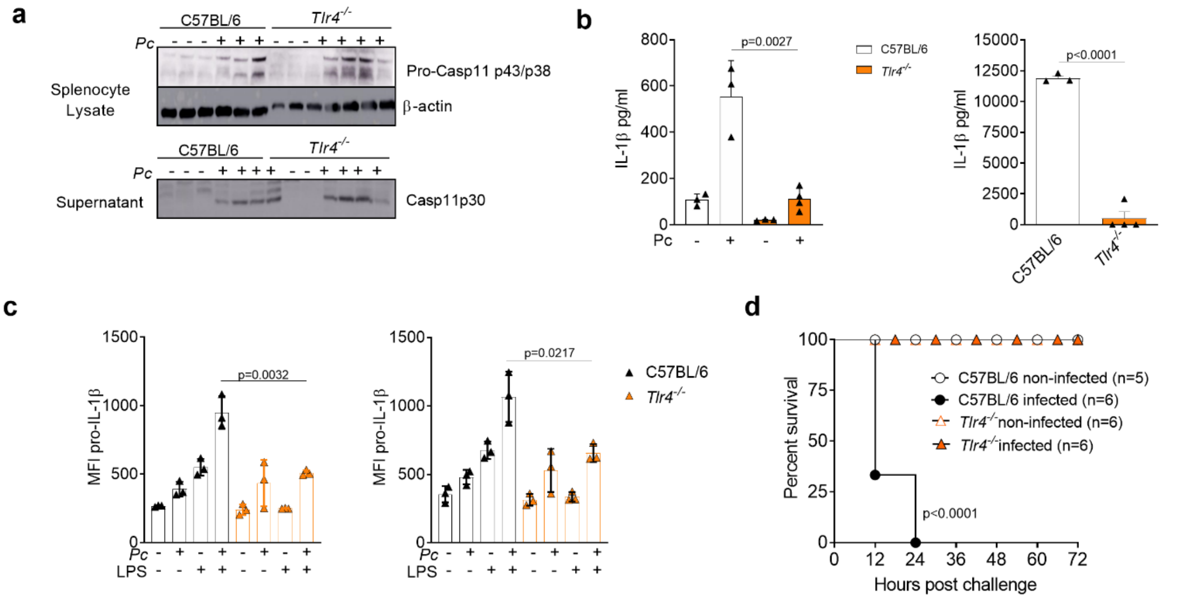

Supplementary Figure 1: TLR4-induced expression of pro-IL1β in *Pc*-infected mice challenged with LPS. All results presented in this figure were obtained from C57BL/6 and *Tlr4*$^{-/-}$ mice either uninfected or at 8 days after infection with *P. chabaudi*, as indicated. **(a)** Splenocytes from mice were lysed with RIPA buffer and analyzed by Western blot using an anti-caspase-11 antibody. Supernatants obtained from splenocyte centrifugation at 400 x g for 5 minutes were analyzed by Western blot using an anti-caspase-11 antibody. Blot employing anti-β-actin was used as loading controls. (**b-left panel**) Splenocytes of uninfected and infected mice were stimulated *in vitro* with LPS (1μg/ml) and IL-1β levels measured by ELISA in 24 hours culture supernatants. (**b-right panel**) Mice were inoculated i.v. with 10 μg/mouse of LPS and plasma collected to measure the levels of circulating IL-1β at 8 hours post-challenge. For all groups n=3 except for infected *Tlr4*$^{-/-}$ n=4. **(c)** Splenocytes from uninfected and infected C57BL/6 and *Tlr4*$^{-/-}$ mice were stimulated with 1 μg/mL of LPS and after two hours used for intracellular staining of pro-IL1β. The pro-IL1β MFI quantification was assayed in live (Live/Dead^{-}) monocytes (CD11b^{+}F4/80^{+}, left panel) and monocyte-derived dendritic cells (CD11b^{+}F4/80^{+}CD11c^{+}MHC II^{+}, right panel) by flow cytometry. For all groups n=3. **(d)** Non-infected and infected mice were challenged with 10 μg/mouse of LPS and followed for survival. In a is a representative blot of two different experiments. In b-d mean ± s.e.m are representatives of two-three different experiments; statistical analysis by unpaired parametric Student's t test. Survival curve was analyzed by log-rank test.

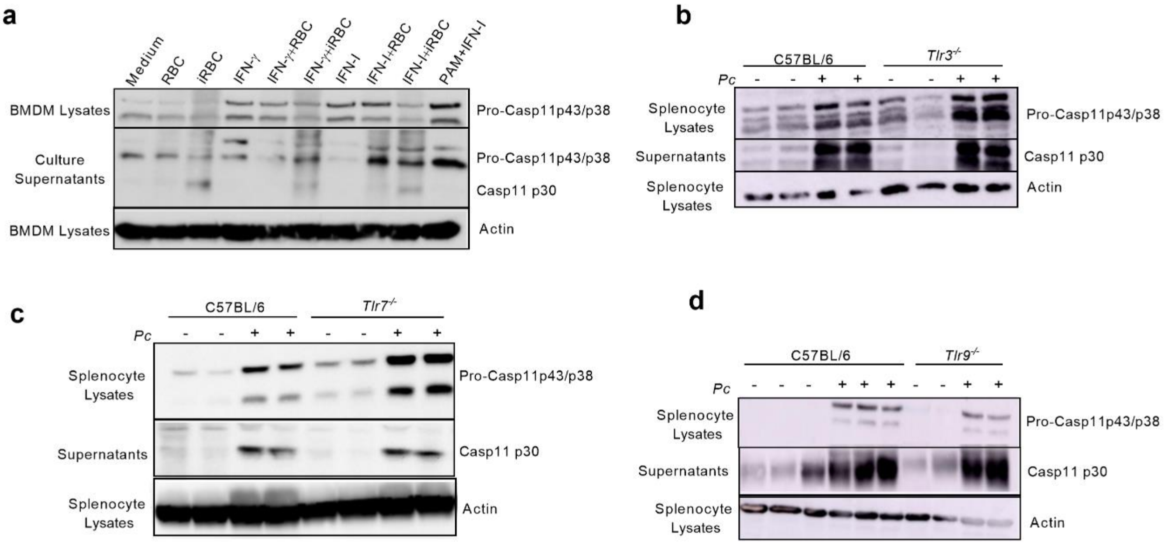

Supplementary Figure 2: Expression of Caspase-11 in *Pc*-infected mice requires IFN-γ and NAS-TLRs. (a) Bone marrow derived macrophages (BMDMs) were cultured in the presence or absence of IFNγ (40 ng/ml), IFN-I (500 U/ml), uninfected red blood cells (RBCs) and/or *Pc*-infected RBCs (iRBCs). Sixteen hours after stimulation, the BMDMs were lysed with RIPA buffer, supernatants collected for protein precipitation and analyzed by Western blot with an anti-caspase-11 antibody. On days 0 (uninfected) and 8 post-infection splenocyte lysates and supernatants obtained from C57BL/6 and **(b)** *Tlr3*$^{-/-}$, **(c)** *Tlr7*$^{-/-}$ as well as **(d)** *Tlr9*$^{-/-}$ mice were analyzed by Western blot using an anti-caspase-11 antibody. Blots employing anti-β-actin were used as loading controls. In a-d, blots are representative of 2 different experiments.

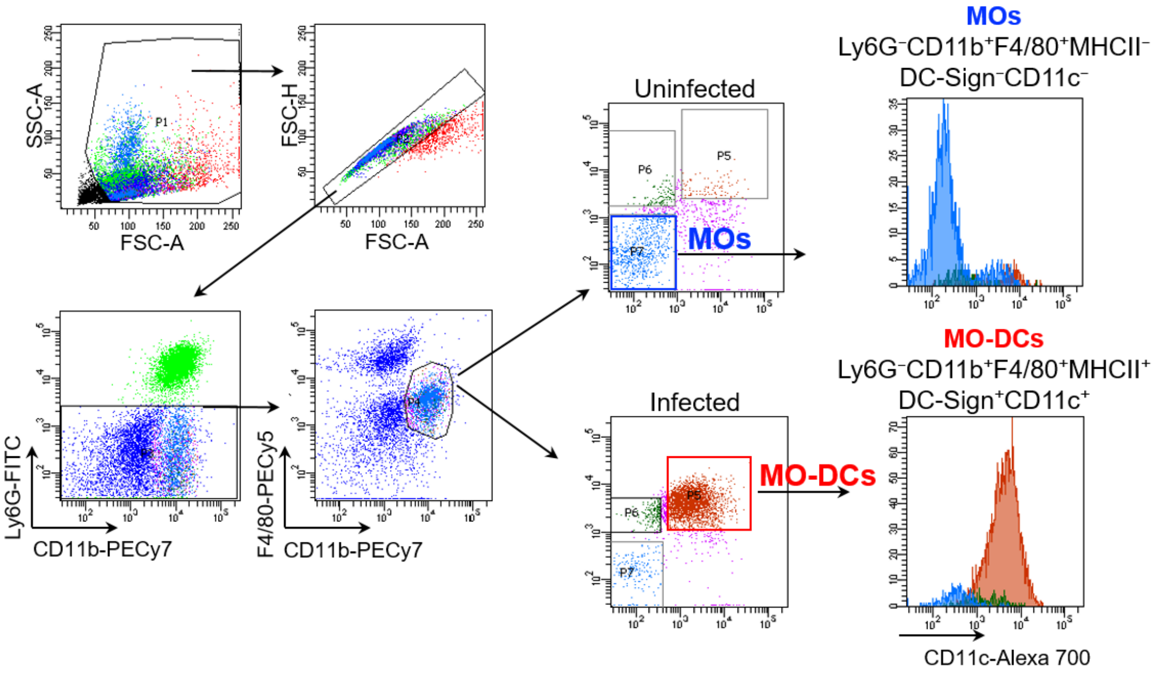

Supplementary Figure 3: Gating Strategy for the analysis of splenic MOs and MO-DCs. Splenocytes were pulled from C57BL/6 mice and isolation of specific populations was performed. MOs (Ly6G−CD11b+F4/80+DC-SIGN-MHCII−) were isolated from uninfected mice and MO-DCs (Ly6G−CD11b+F4/80+DC-Sign+MHCII+) were isolated from infected mice. All MFI data from the manuscript were performed in MOs or MO-DCs using the gating strategies showed in this figure.

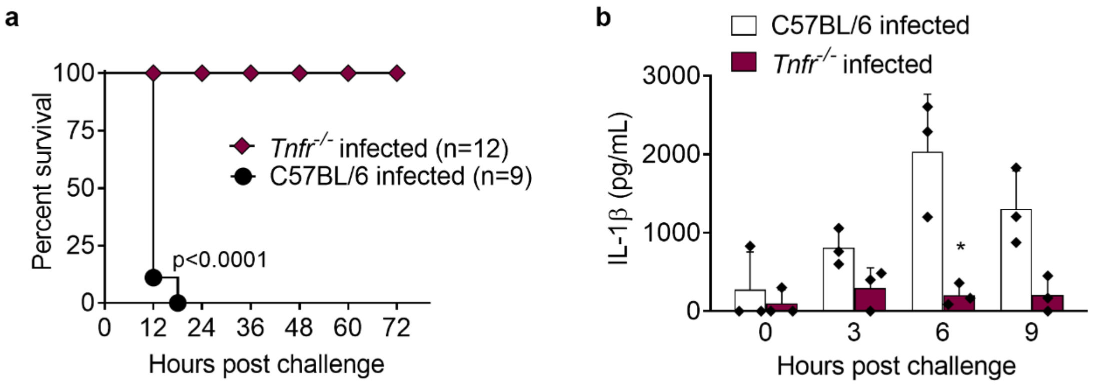

Supplementary Figure 4: Impaired IL-1β release in *Pc*-infected *Tnfr*$^{-/-}$ mice challenged with LPS. (a) Non infected controls and *Pc*-infected C57BL/6 and *Tnfr*$^{-/-}$ mice were challenged with 10 μg/mouse of LPS and followed for survival. **(b)** C57BL/6 (n=3) and *Tnfr*$^{-/-}$ (n=3) mice were inoculated i.v. with 10 μg/mouse of LPS and plasma collected before (time 0), 3, 6 and 9 hours after LPS inoculation to measure the levels of circulating IL-1β by ELISA. All data is representative of two-different experiments. Survival curve was analyzed by log-rank test. In b**,** statistical analysis by two-way ANOVA. * p=0.01.

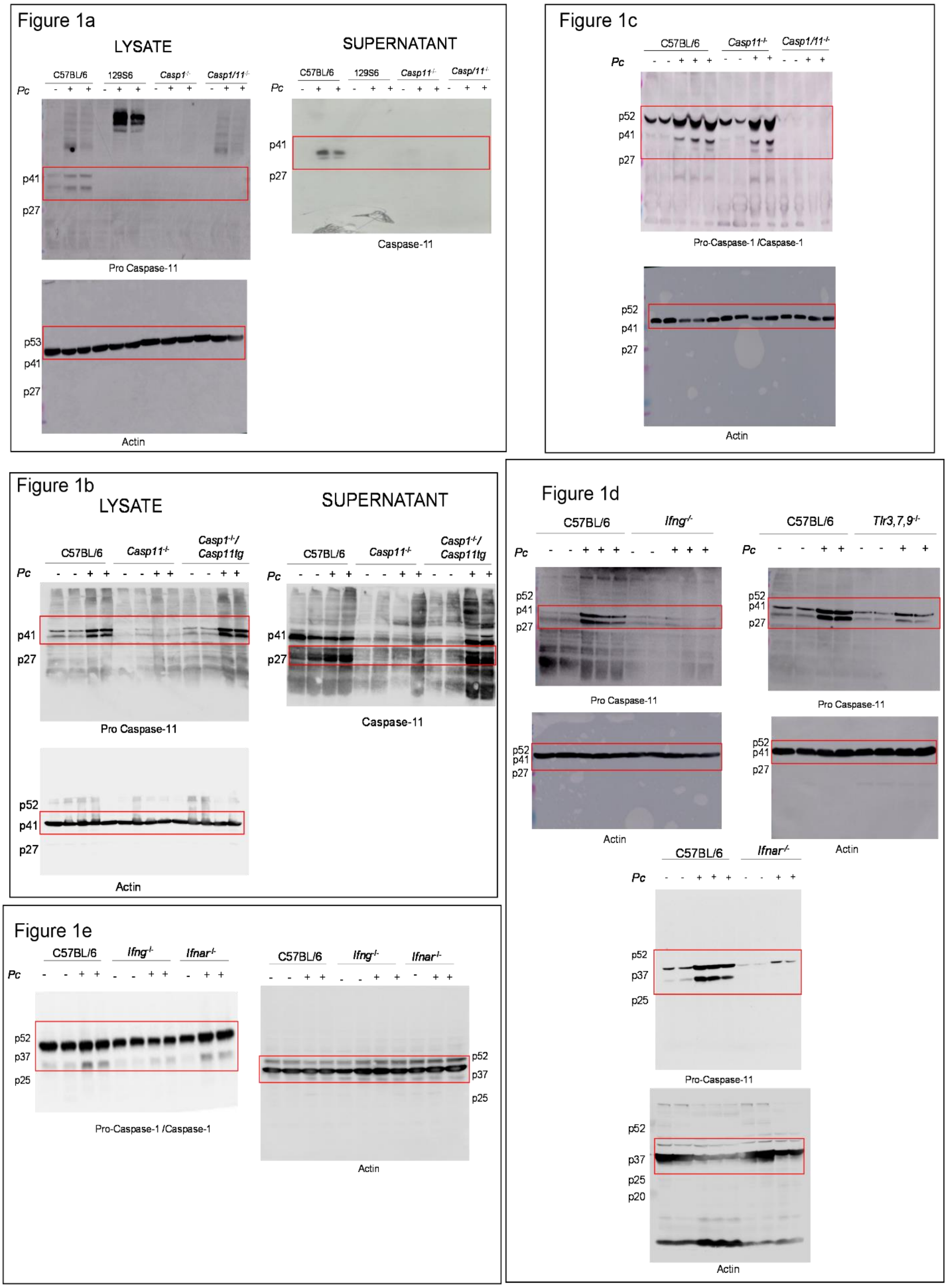

Supplementary Figure 5: Uncropped Western blots from Figure 1. Membranes were probed using the antibodies against indicated proteins (anti caspase-1, anti-caspase-11, anti-actin).

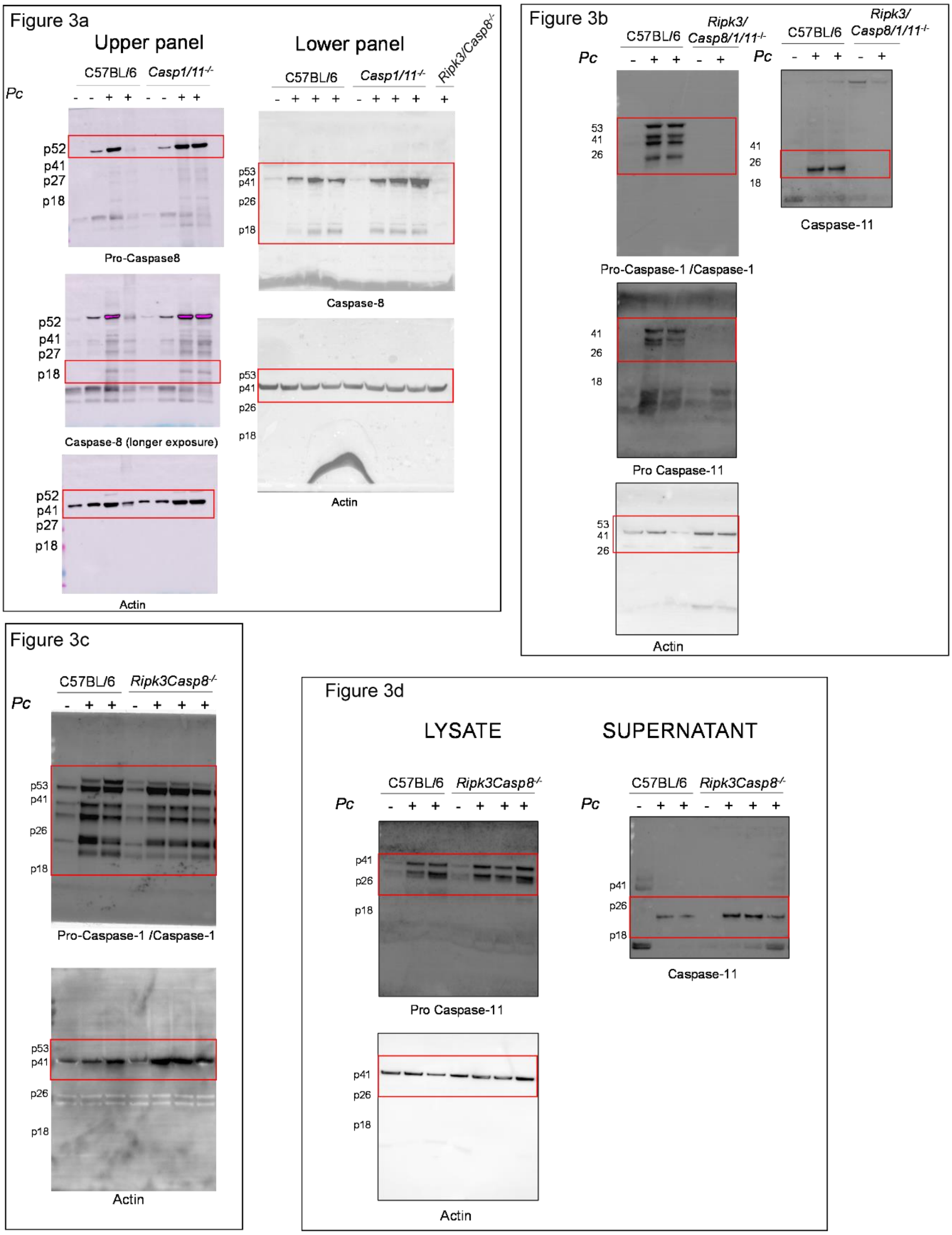

Supplementary Figure 6: Uncropped Western blots from Figure 3. Membranes were probed using the antibodies against indicated proteins (anti-caspase-8, anti-cleaved-caspase-8, anti-caspase-1, anti-caspase-11, anti-actin).

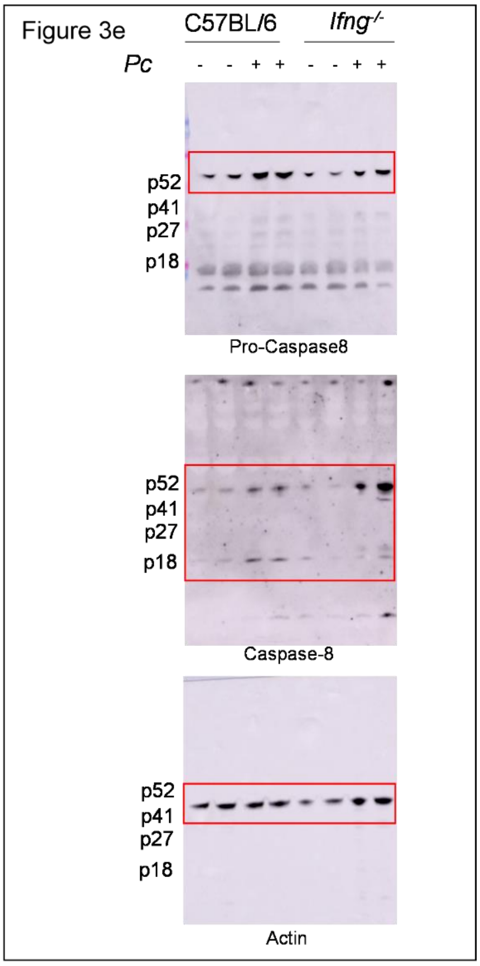

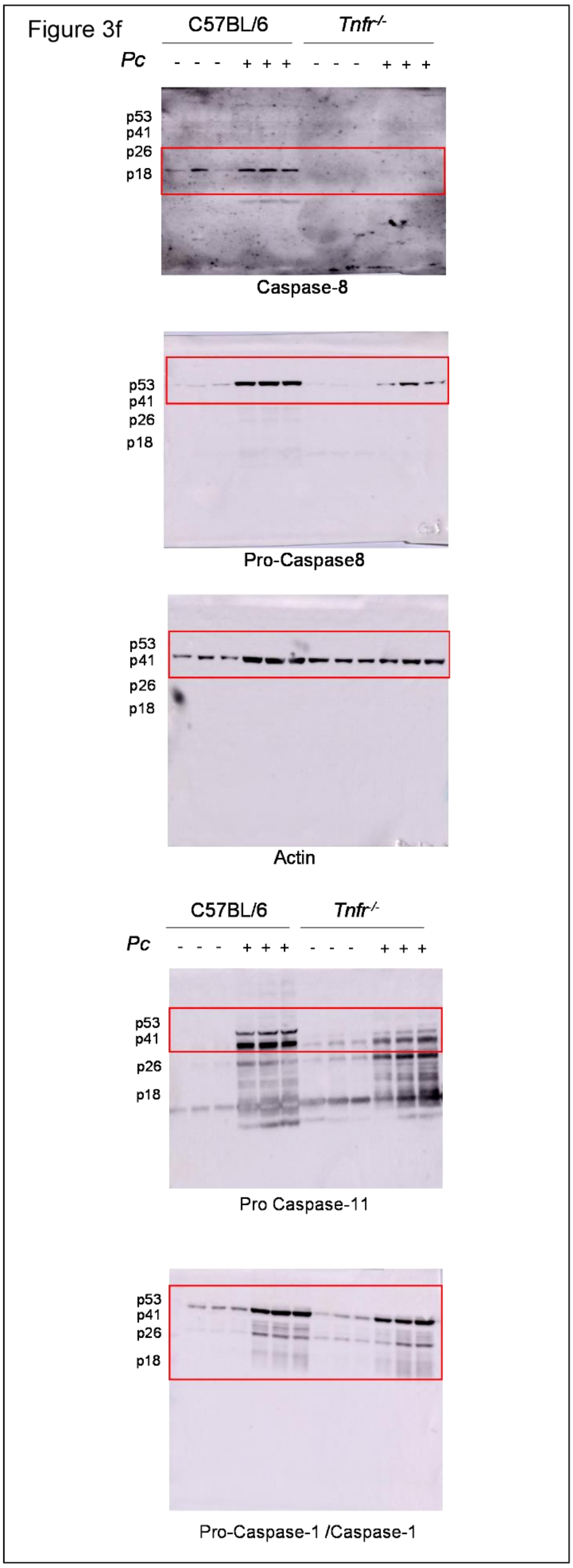

Supplementary Figure 7: Uncropped Western blots from Figure 3. Membranes were probed using the antibodies against indicated proteins (anti-caspase-8, anti-cleaved-caspase-8, anti-caspase-1, anti-caspase-11, anti-actin).

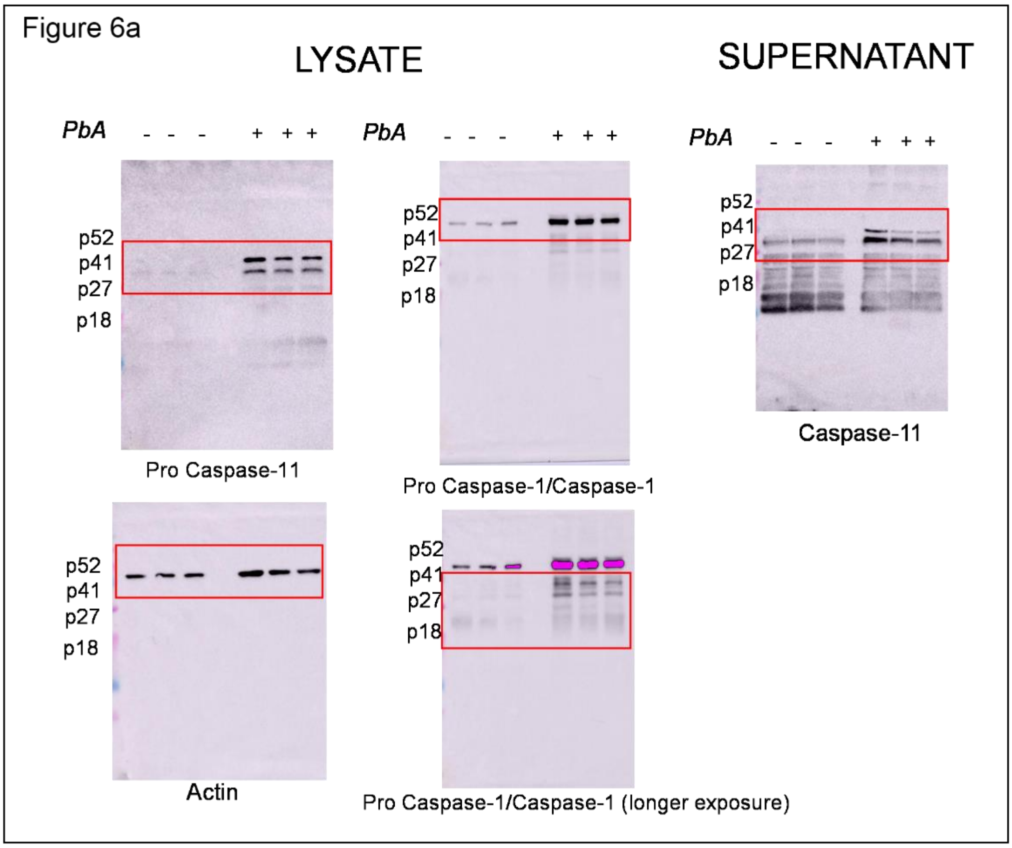

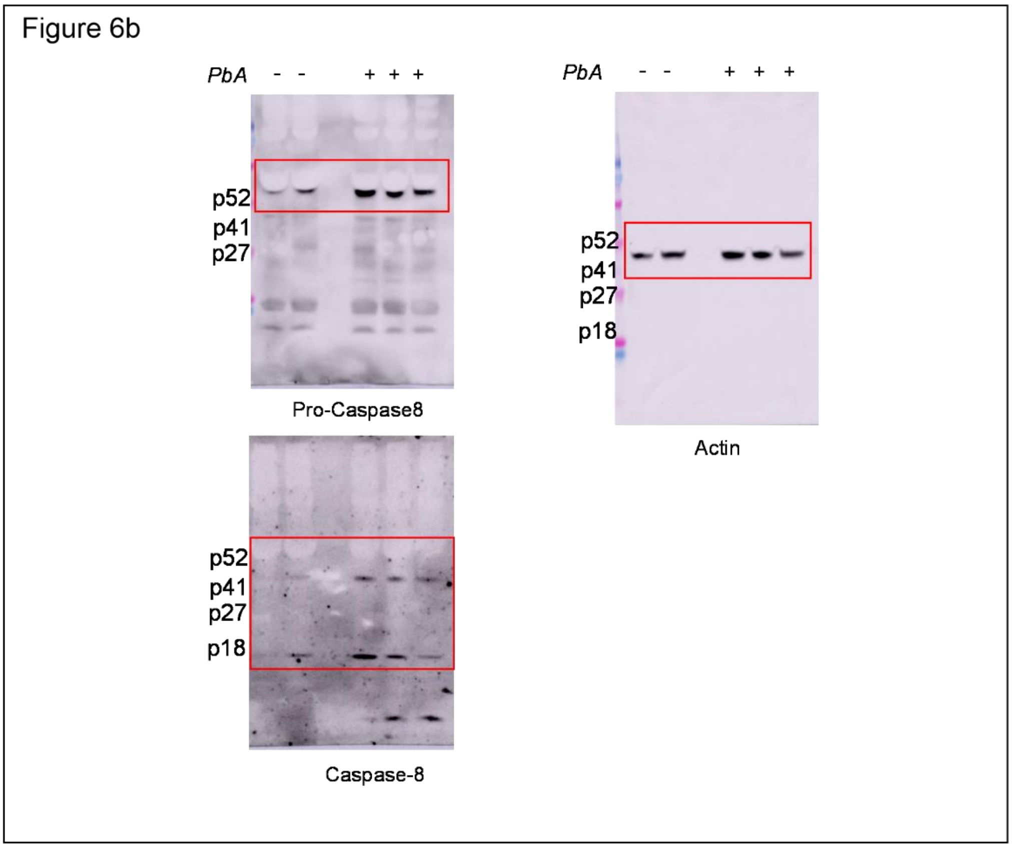

Supplementary Figure 8: Uncropped Western blots from Figure 6. Membranes were probed using the antibodies against indicated proteins (anti-caspase-8, anti-cleaved-caspase-8, anti-caspase-1, anti-caspase-11, anti-actin).

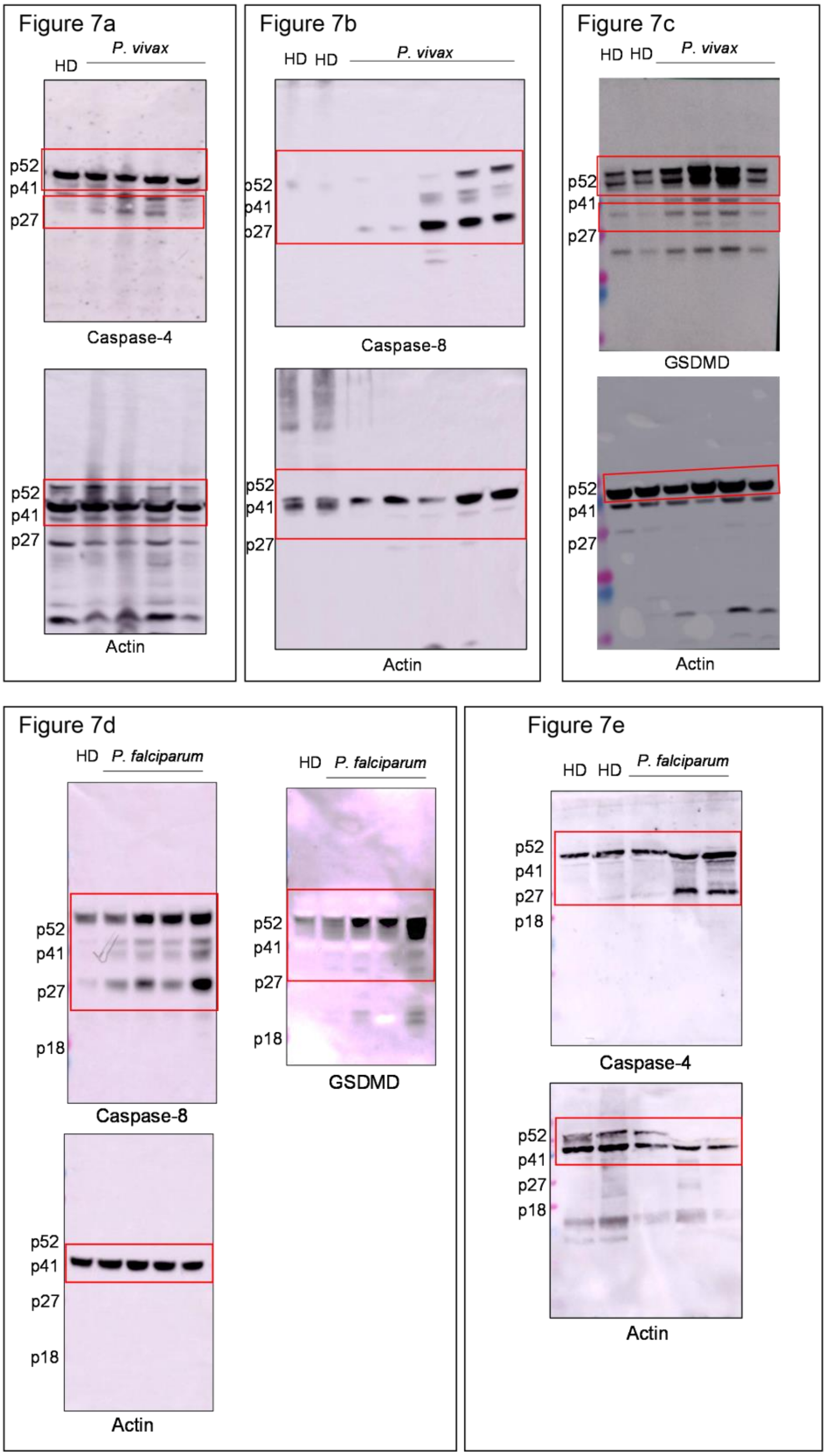

Supplementary Figure 9: Uncropped Western blots from Figure 7. Membranes were probed using the antibodies against indicated human proteins (anti-caspase-4, anti-caspase-8, anti-GSDMD, anti-actin).

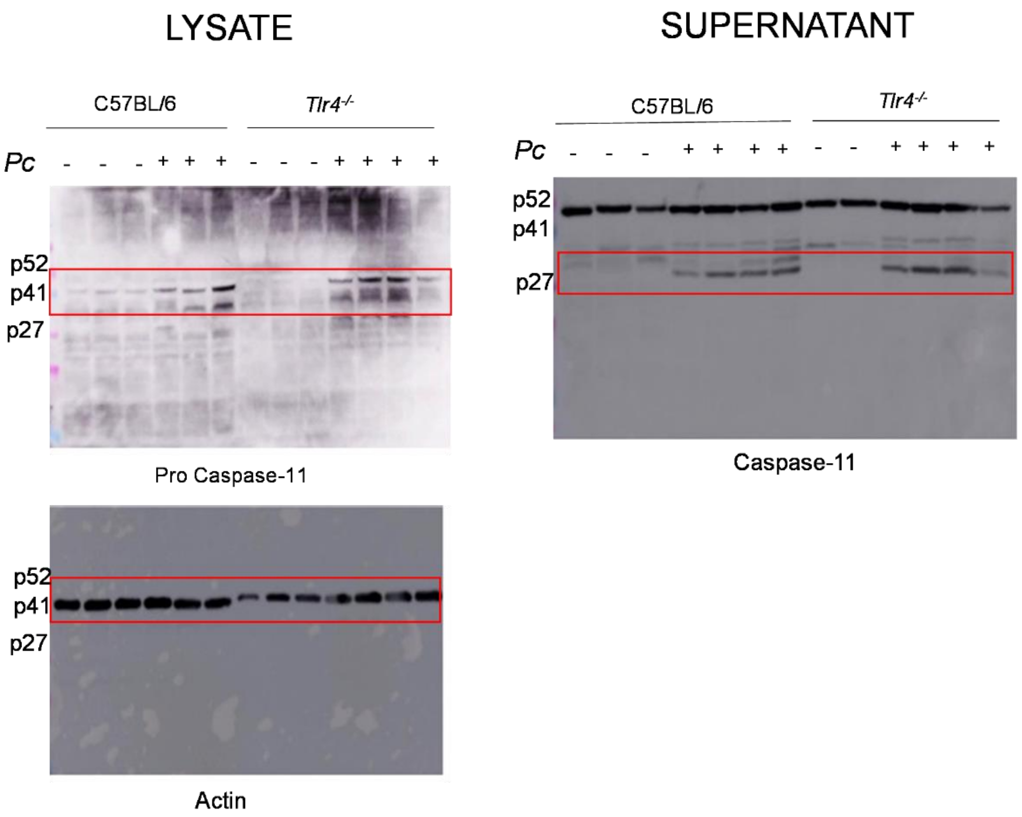

Supplementary Figure 10: Uncropped Western blots from Supplementary Figure 1. Membranes were probed using the antibodies against indicated proteins (anti-caspase-11, anti-actin).

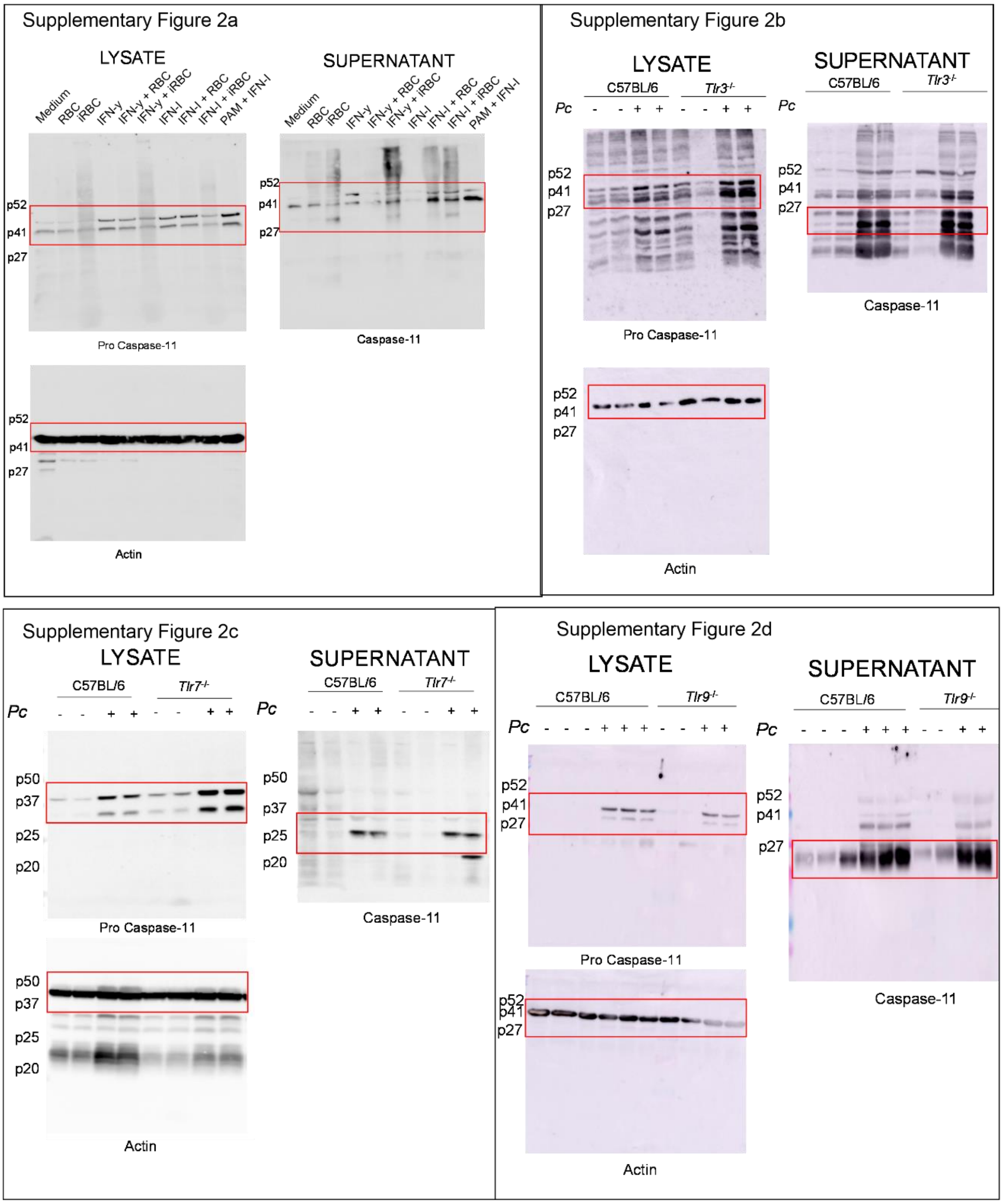

Supplementary Figure 11: Uncropped Western blots from Supplementary Figure 2. Membranes were probed using the antibodies against indicated proteins (anti-caspase-11, anti-actin).

Supplementary Table 1: List of reagents and software

Reagent or Resource	Source	Identifier
Anti-Casp4	Cell Signaling	Cat# 4450S RRID:AB_1950386
Anti-Casp8 (Human) (Clone 12F5)	Enzo Lifescience	Cat# ALX-804-242-C100 RRID:AB_2050949
Anti-Casp8 (Mouse) (Clone 1G12)	Enzo Lifescience	Cat# ALX-804-447-C100 RRID:AB_2050952
Anti-GSDM-D (Human) (Clone 126-138)	Sigma	Cat# G7422 RRID:AB_1850381
Anti-Casp1 (Clone Casper-1)	Adipogen	Cat# AG-20B-0042 RRID:AB_2490248
Anti Casp11 (Clone 17D9)	Novus Biologicals	Cat# NB120-10454 RRID:AB_2259600
Anti-Cleaved Casp8 (Clone D5B2)	Cell Signaling	Cat# 8592 RRID:AB_10891784
Anti-Actin	Sigma	Cat# A2066 RRID:AB_476693
Anti-Mouse (HRP)	Jackson Immunoresearch	Cat# 115-035-003 RRID:AB_10015289
Anti-Rabbit (HRP)	Jackson Immunoresearch	Cat# 111-035-144 RRID:AB_2307391
Anti-Rat (HRP)	Jackson Immunoresearch	Cat# 112-035-175, RRID:AB_2338140
Anti-CD11b Pecy7 (Clone M1/70)	eBioscience	Cat# 25-0112-82 RRID:AB_469588
Anti-F4/80 APC (Clone BM8)	Biolegend	Cat# 123116 RRID:AB_893481
Anti-F4/80 PECy5 (Clone BM8)	eBioscience	Cat# 15-4081-82 RRID: AB_468798
Anti-CD11c AF700 (Clone N418)	Biolegend	Cat# 117320 RRID:AB_528736
Anti-MHCII Apccy7 (Clone M5/114.15.2)	Biolegend	Cat# 107628 RRID:AB_2069377
Anti-Pro-IL-1β FITC (Clone NJTEN3)	eBioscience	Cat# 11-7114-82 RRID:AB_10718251
Anti-Ly6G FITC (clone 1A8)	eBioscience	Cat# 11-9668-82 RRID AB_2572532
Anti-DC-SIGN (clone MMD3)	eBioscience	Cat# 50-2094-82 RRID AB_11219065
CD14 microbeads	Miltenyi Biotec	Cat# 130-050-201

CD11b microbeads	Miltenyi Biotec	Cat# 130-049-601
Mouse IL-1β ELISA	ThermoFisher	Cat# 88-7013-88
Mouse TNFα ELISA	ThermoFisher	Cat# 88-7346-88
Recombinant mouse IFNγ	Sigma Aldrich	Cat# I4777
Recombinant type I IFN	PBL Assay Science	Cat# 11200-2
LPS O55:B55 From *E. Coli*	Sigma Aldrich	Cat# L2880
Ultrapure LPS, E. coli 0111:B4	InvivoGen	Cat# tlrl-3pelps
Ficoll-Paque Plus	GE Healthcare	Cat# 17-1440-02
RPMI	Corning	Cat# 10-040-CV
DMEM	Corning	Cat# 10-01300CV
Fetal Bovine Serum	ThermoFisher	Cat# 16000-044
Penicillin And Streptomycin	Corning	Cat# 30-001-CI
PBS	Corning	Cat# 21-040-CM
RIPA Buffer	Sigma	Cat# R0278
HALT Protease inhibitor cocktail	ThermoFisher	Cat# PI78443
Clarity Max Western ECL Substrate	BioRad	Cat# 1705062
Qiagen RNeasy Mini Kit	Qiagen	Cat#74104
Qiagen Rnase-Free Dnase Set	Qiagen	Cat# 79254
TruSeq Stranded mRNA Kit	Illumina	Cat# 15032612
AmCyan Live/Dead Cell Viability Assay	ThermoFisher	Cat# L34957
Flowjo	Flowjo	Http://Www.Flowjo.Com/
Excel	Microsoft	Http://Www.Products.Office.Com/En-Us/Excel
Graphpad Prism 7.0 Software	Graphpad Software	Http://Www.Graphpad.Com/Scientificsoftware/Prism/
The R project for statistical computing	R Foundation	https://www.r-project.org/

Trimmomatic	Bolger et al., 2014.[1]	http://www.usadellab.org/cms/?page=trimmomatic
CUFFLINKS	Trapnell et al., 2012. [2]	http://cole-trapnell-lab.github.io/cufflinks/
STAR aligner	Dobin et al., 2013. [3]	STAR aligner
Ingenuity Pathway Analysis	Qiagen	https://www.qiagenbioinformatics.com/products/ingenuity-pathway-analysis/

Supplementary references

1. Bolger AM, Lohse M, Usadel B. Trimmomatic: a flexible trimmer for Illumina sequence data. *Bioinformatics* **30(15)**, 2114-2120 (2014).

2. Trapnell, C., Roberts, A., Goff, L. *et al.* Differential gene and transcript expression analysis of RNA-seq experiments with TopHat and Cufflinks. *Nat Protoc* **7**, 562–578 (2012).

3. Dobin A, et al. STAR: ultrafast universal RNA-seq aligner. *Bioinformatics* **29**, 15–21 (2013).
