# Supplementary material for: Caspase-8 mediates inflammation and disease in rodent malaria
Source: Nat Commun. 2020 Sep 14;11:4596. doi: 10.1038/s41467-020-18295-x (PMC7490701; doi:10.1038/s41467-020-18295-x)
Supplement: Supplementary file 3 — Reporting Summary [file 41467_2020_18295_MOESM3_ESM.pdf]

nature research

Corresponding author(s): Ricardo T. Gazzinelli (ricardo.gazzinelli@umassmed.edu)

Last updated by author(s): Aug 2, 2020

Reporting Summary

Nature Research wishes to improve the reproducibility of the work that we publish. This form provides structure for consistency and transparency in reporting. For further information on Nature Research policies, see our Editorial Policies and the Editorial Policy Checklist.

Statistics

For all statistical analyses, confirm that the following items are present in the figure legend, table legend, main text, or Methods section.

n/a	Confirmed	
☐	☒	The exact sample size (*n*) for each experimental group/condition, given as a discrete number and unit of measurement
☐	☒	A statement on whether measurements were taken from distinct samples or whether the same sample was measured repeatedly
☐	☒	The statistical test(s) used AND whether they are one- or two-sided<br>*Only common tests should be described solely by name; describe more complex techniques in the Methods section.*
☐	☒	A description of all covariates tested
☐	☒	A description of any assumptions or corrections, such as tests of normality and adjustment for multiple comparisons
☐	☒	A full description of the statistical parameters including central tendency (e.g. means) or other basic estimates (e.g. regression coefficient) AND variation (e.g. standard deviation) or associated estimates of uncertainty (e.g. confidence intervals)
☐	☒	For null hypothesis testing, the test statistic (e.g. *F*, *t*, *r*) with confidence intervals, effect sizes, degrees of freedom and *P* value noted<br>*Give P values as exact values whenever suitable.*
☒	☐	For Bayesian analysis, information on the choice of priors and Markov chain Monte Carlo settings
☒	☐	For hierarchical and complex designs, identification of the appropriate level for tests and full reporting of outcomes
☒	☐	Estimates of effect sizes (e.g. Cohen's *d*, Pearson's *r*), indicating how they were calculated

Our web collection on statistics for biologists contains articles on many of the points above.

Software and code

Policy information about availability of computer code

Data collection	Western Blot Images: ImageLab™ Touch Software V6.0.1 (Bio-Rad)<br>Flow Cytometry: BD FACSDIVA V8.0.1<br>ELISA:SOFTmaxPRO V4.3.1 LS
Data analysis	Microsoft Excel 2006<br>Western Blot Images: ImageStudio™ V5.2<br>Flow Cytometry: FlowJo V10.5.3<br>RNA-Seq: -Trimmomatic V0.32<br>-STAR aligner V2.4.0.1<br>-Cufflinks V2.2.1<br>-IPA – Ingenuity Pathway Analysis V01-12 (Qiagen)<br>-Cytoscape V3.7.1<br>Statistics: -R Project for Statistical Computing V3.6.2(https://www.r-project.org/)<br>-GraphPad Prism V7.0c

For manuscripts utilizing custom algorithms or software that are central to the research but not yet described in published literature, software must be made available to editors and reviewers. We strongly encourage code deposition in a community repository (e.g. GitHub). See the Nature Research guidelines for submitting code & software for further information.

Data

Policy information about availability of data

All manuscripts must include a data availability statement. This statement should provide the following information, where applicable:

- Accession codes, unique identifiers, or web links for publicly available datasets
- A list of figures that have associated raw data
- A description of any restrictions on data availability

The data sets generated and analyzed during the current study are available in the GEO-Gene Expression Omnibus (NCBI) repository access code GSE126381, [https://www.ncbi.nlm.nih.gov/geo/query/acc.cgi?acc=GSE126381]. The manuscript contains a data availability statement with this code and indication to accessing raw data.

Field-specific reporting

Please select the one below that is the best fit for your research. If you are not sure, read the appropriate sections before making your selection.

[x] Life sciences [ ] Behavioural & social sciences [ ] Ecological, evolutionary & environmental sciences

For a reference copy of the document with all sections, see nature.com/documents/nr-reporting-summary-flat.pdf

Life sciences study design

All studies must disclose on these points even when the disclosure is negative.

Sample size	The group sizes for experiments with mice or human samples were determined by power calculations statistical analysis.
Data exclusions	Negative and positive controls have worked properly so no data were excluded from the analysis.
Replication	The experiments were repeated at least twice, if the statistical significance was P<0.05. In case we have find a trend but the data didn't reach a statistical significance, the experiment was repeated a third time. All attempts at replication were successful.
Randomization	In both human and mouse experiments we used age and sex-matched individual in the control groups.
Blinding	Except for the clinical score that was somewhat subjective; which performed by two independent observers, we didn't find the need for blinding analysis in the other experiments because the phenotypes were very distinct.

Reporting for specific materials, systems and methods

We require information from authors about some types of materials, experimental systems and methods used in many studies. Here, indicate whether each material, system or method listed is relevant to your study. If you are not sure if a list item applies to your research, read the appropriate section before selecting a response.

Materials & experimental systems

n/a	Involved in the study	
[ ]	[x]	Antibodies
[x]	[ ]	Eukaryotic cell lines
[x]	[ ]	Palaeontology and archaeology
[ ]	[x]	Animals and other organisms
[ ]	[x]	Human research participants
[x]	[ ]	Clinical data
[x]	[ ]	Dual use research of concern

Methods

n/a	Involved in the study	
[x]	[ ]	ChIP-seq
[ ]	[x]	Flow cytometry
[x]	[ ]	MRI-based neuroimaging

Antibodies

Antibodies used

-Anti-Casp4 Cell Signaling Cat# 4450S RRID:AB_1950386
-Anti-Casp8 (Human) (Clone 12F5) Enzo Lifescience Cat# ALX-804-242-C100 RRID:AB_2050949
-Anti-Casp8 (Mouse) (Clone 1G12) Enzo Lifescience Cat# ALX-804-447-C100 RRID:AB_2050952
-Anti-Gsdmd (Human) (Clone 126-138) Sigma Cat# G7422 RRID:AB_1850381
-Anti-Casp1 (Clone Casper-1) Adipogen Cat# AG-20B-0042 RRID:AB_2490248
-Anti Casp11 (Clone 17D9) Novus Biologicals Cat# NB120-10454 RRID:AB_2259600
-Anti-Cleaved Casp8 (Clone D5B2) Cell Signaling Cat# 8592 RRID:AB_10891784
-Anti-Actin Sigma Cat# A2066 RRID:AB_476693
-Anti-Mouse (HPR) Jackson Immunoresearch Cat# 115-035-003 RRID:AB_10015289

	-Anti-Rabbit (HPR) Jackson Immunoresearch Cat# 111-035-144 RRID:AB_2307391<br>-Anti-Rat (HRP) Jackson Immunoresearch Cat# 112-035-175, RRID:AB_2338140<br>-Anti-CD11b Pecy7 (Clone M1/70) eBioscience Cat# 25-0112-82 RRID:AB_469588<br>-Anti-F4/80 APC (Clone BM8) Biolegend Cat# 123116 RRID:AB_893481<br>-Anti-F4/80 PECy5 (Clone BM8) eBioscience Cat# 15-4081-82 RRID: AB_468798<br>-Anti-CD11c AF700 (Clone N418) Biolegend Cat# 117320 RRID:AB_528736<br>-Anti-MHCII APCcy7 (Clone M5/114.15.2) Biolegend Cat# 107628 RRID:AB_2069377<br>-Anti-Pro-IL-1B FITC (Clone NJTEN3) eBioscience Cat# 11-7114-82 RRID:AB_10718251<br>-Anti-Ly6G FITC (clone 1A8) eBioscience Cat# 11-9668-82 RRID AB_2572532<br>-Anti-DC-SIGN E-Fluor 660 (clone MMD3) eBioscience Cat# 50-2094-82 RRID AB_11219065
Validation	All antibodies were commercial and validated by the manufacturer. The antibodies for flow cytometry have been tested for mouse splenocytes and were properly titrated before the experiments. The dilutions of antibodies used for Western Blot were recommended by the manufacturer and optimized in our experiments.

Animals and other organisms

Policy information about studies involving animals; ARRIVE guidelines recommended for reporting animal research

Laboratory animals	All mouse lineages used in this study have been backcrossed for at least 10 generations into the C57BL/6 genetic background. Caspase-11-/-, Caspase-1-/-/11tg and GSDM-D-/- were provided by Dr. Vishva Dixit from Genentech (San Francisco, CA). Caspase-1-/-/11-/- and TLR3-/- mice were provided by Dr. Richard Flavell from Yale University (New Haven, CT). The Caspase 1-/- mice were provided by Dr. Devi Kanneganti Thirumala from St. Jude Children's Research Hospital (Memphis, TN). RIP3-/-, Caspase 8-/-RIP3-/- and Caspase 1-/-11-/-8-/-RIP3-/- were provided by Egil Lien. The TLR9-/-, TLR4-/- and TLR7-/- mice were provided by Dr. Shizuo Akira from Osaka University (Osaka, Japan). The RIP3-/-caspase-8-/-GSDM-D-/- and TLR3/7/9-/- mice were generated in our laboratory by genetic crosses. The C57BL/6, IFN-γ-/-, TNFR-/-, IFNABR and 129S6 were originally obtained from Jackson Labs. All mouse lineages mentioned above were bred and maintained in microisolators at Fiocruz-Minas and UMMS on a 12h dark/light cycle, temperature range was 68°F - 79°F and humidity between 30-70%. Female and male mice between 6 to 10 weeks old were used in all experiments.
Wild animals	The study did not involve wild animals.
Field-collected samples	The study did not include samples collected from the field.
Ethics oversight	Experiments with mice were conducted according to institutional guidelines for animal ethics and approved by the institutional ethic committees from Oswaldo Cruz Foundation (Fiocruz-Minas, CEUA/LW15/14 and LW16/18) and UMMS (IACUC/A-2371-15), respectively.

Note that full information on the approval of the study protocol must also be provided in the manuscript.

Human research participants

Policy information about studies involving human research participants

Population characteristics	As approved in our ethical committees the patients were older then 16 years of age and no older then 60 years. We used both male and female. In Brazil there is a great diversity in genetic origin, reflecting the make-up of our work place, and includes African Americans, Asians, South American Indians, Mestizos and Caucasians of European origin.
Recruitment	A total of 9 P. vivax-infected and 6 P. falciparum-infected patients with uncomplicated malaria were enrolled in this study at Centro de Pesquisa de Medicina Tropical de Rondonia (CEPEM) in Porto Velho, Rondonia, a malaria endemic area in the Amazon region of Brazil. According to the World Health Organization, uncomplicated malaria is a symptomatic infection with malaria parasitemia without signs of severity and/or evidence of vital organ dysfunction. Healthy donors volunteers (n=8) from Porto Velho or Belo Horizonte were used as negative controls. All volunteers were randomly recruted so that any biased results were minimized.
Ethics oversight	The Ethical Committee on Human Experimentation from Centro de Pesquisas em Medicina Tropical (CEP-CEPEM 096/2009), the Brazilian National Ethical Committee (CONEP 15653) from Ministry of Health and the Institutional Review Board from the University of Massachusetts Medical School (UMMS, IRB-ID11116) approved the present study performed with malaria patients.

Note that full information on the approval of the study protocol must also be provided in the manuscript.

Flow Cytometry

Plots

Confirm that:

- [x] The axis labels state the marker and fluorochrome used (e.g. CD4-FITC).
- [x] The axis scales are clearly visible. Include numbers along axes only for bottom left plot of group (a 'group' is an analysis of identical markers).
- [x] All plots are contour plots with outliers or pseudocolor plots.
- [x] A numerical value for number of cells or percentage (with statistics) is provided.

Methodology

Sample preparation	Splenocytes from uninfected and infected (day8) mice were collected. Cell suspensions where prepared by smashing the spleens through a cell strainer and treatment with a RBC lysis buffer. The total cells obtained where counted and 2x10^6 cells were plated in RPMI+ Brefeldin (5ug/mL) with ou without LPS (1ug/mL) and incubated for 2hours. After incubation, the cells were collected and first incubated with a viability fluorescent dye (AmCyam) and then stained with a cocktail of antibodies: CD11cAlexa fluor700, CD11b-PEcy7, MHCII-PE, F4/80- APC and pro-IL-1β-FITC.<br>For Cell Sorting experiments spleens from uninfected and PbA-infected mice, at 5 day post infection, were harvested and processed as described above. The cell suspension was then incubated with a cocktail of antibodies: Ly6G-FITC, CD11b-PE-Cy7, F4/80-PE-Cy5, CD11c-Alexa 700, MHC II-APCCy7, DCSIGN-APC e-fluor 660, CD11c-Alexa Fluor700 and acquired and sorted as Monocytes from uninfected samples: Ly6G-CD11b+F4/80+DCSIGN-MHCII-CD11c- or MODCs from infected mice: Ly6G-CD11b+F4/80+DCSIGN+MHCII+CD11c+.
Instrument	Flow Cytometry was performed on the LSR II (BD Biosciences) and cell sorting on the BD FACS Aria II.
Software	Collection of data was performed using the Digital DIVA hardware and software and analysis using FLowJo software v.10.
Cell population abundance	Monocytes represents approximately 1-2% of total splenocytes and MODCs during Plasmodium infection are approximately 60% of total splenic monocytes. Dendritic cells on the other hand are approximately 5% on Plasmodium infected spleens. Purity of sorted cells was evaluated by acquisition of a fraction of the sample post-sorting and we observed purity higher then 95% in all samples.
Gating strategy	For pro-IL-1B intracellular staining cells where gated on FSC-HxFSC-A to exclude doublets, then total cells where gated according to SSC-AxFSC-A. Next dead cells where excluded according to positivity for the live/dead assay and live cells where cells gated as CD11b+F4/80+ (monocytes) or CD11c+MHCII+ (dendritic cells). The MFI for FITC fluorescence (pro-IL-1B) was then measured to correlate with the expression level of these protein.<br>For cell sorting experiments: cells where gated on FSC-HxFSC-A to exclude doublets, then total cells where gated according to SSC-AxFSC-A. Next Ly6G+ cells where excluded and CD11b+F4/80+ where gated. These positive cells where gated as DCSIGNMHCII- on uninfected samples and DCSIGN+MHCII+ on infected samples. In both cases we confirmed the absence or presence of CD11c, respectevely.

- [x] Tick this box to confirm that a figure exemplifying the gating strategy is provided in the Supplementary Information.

April 2020
